# Reaction against Hysterectomy

**Published:** 1902-10-18

**Authors:** 


### Hospital Clinics and Medical Progress.

### REACTION AGAINST HYSTERECTOMY.

Dr. Andrew McCosh,[1] of New York, advocates myomectomy in many cases in which at present hysterectomy is commonly employed. During the past ten or twelve years, he says, the surgical treatment of the pelvic organs of the female has happily become more preservative, or perhaps one might term it less destructive. Previous to this time many operators went so far as to argue that if the tubes and ovaries were diseased it was best to remove all the pelvic organs. Now, however, the uterus with its adnexa are but seldom removed where a less radical operation will suffice, and cystic ovaries are not as a rule removed *in toto*, at least the Fallopian tubes are saved in whole or in part. But in cases of fibro-myomata of the uterus less progress has been made in the direction of preservation of the organ. "It is true," he says, "that within the past few years attempts are being made by a few abdominal surgeons to be less sacrificial in their treatment of these cases, but the majority of operators still consider that a uterus which is the seat of injurious fibroid tumours is one which demands removal." He does not, in saying this, refer to cases in which the tumours are small or cause but few symptoms, but to grave cases in which a woman's life or happiness is threatened by excessive hæmorrhages or pain, and he draws the conclusion, from conversation with surgeons, from the proceedings of the societies, and from other sources, that "in the practice of most surgeons myomectomy is but rarely done, its proportion to hysterectomy being about one in ten or even one in twenty." In his own practice six years ago myomectomy bore toward hysterectomy the ratio of 1 to 23; in 1900, the ratio of 5 to 27; and in the past year the ratio of 22 to 17. If the tumours are subserous, moderately small, and not very numerous, the vagina offers a suitable route by which they may be removed. The application of this method is however limited, and it is with abdominal myomectomy that he more especially deals, the patient being placed in the Trudelenburg position. In elderly women the uterus with its tumours is removed, but in younger women a careful examination is made as soon as the uterus has been drawn through the incision to determine whether it is possible to remove all the fibroid tumour, and yet to leave sufficient uterine tissue to form a useful organ. In regard to this question Dr. McCosh is not influenced by the location of the tumours as regards their relation to the uterine canal, the mere fact that the tumour is interstitial or submucous, weighing but little in deciding which operation shall be done. The size and number of the tumours are also more or less disregarded. "There are, of course, certain cases in which myomectomy is either impossible or too dangerous; these are apt to be cases in which the uterus from the cervix to the fundus is infiltrated with scores of small fibroids, perhaps extending into the broad ligament. There are also, of course, cases in which the tumours are of very large size, and in which hysterectomy must be the operation of choice." Tumours weighing six, eight, and ten pounds, however, have been removed, and frequently their number has exceeded twenty.

In performing the operation, if the number of tumours be small, and if they be well isolated from each other, a separate incision is made through the uterine tissue for each. The tumour is then either torn out of its bed or enucleated with the fingers, force being employed to accomplish this rapidly, and, with the exception perhaps of pressure by an assistant's finger upon the edges of the cut muscle, no attempt is made to prevent hæmorrhage until the tumour has been removed. But if the tumours be multiple and widely distributed throughout the uterus, the uterus must be more or less split open from the fundus downward on either its anterior or posterior surface; or it may be necessary to completely divide it into halves as far downward as the inner os, each half remaining attached to its broad ligament; in either case the interior of the uterus is widely exposed to view. With the uterus thus laid open every part can be readily palpated, and tumours of very small size can be easily felt. "Careful search is made by palpation of the uterine wall between the finger and thumb, and all tumours, no matter how minute, are removed by section through the overlying muscular tissue, either from the exterior or the interior." Interstitial tumours are seldom single, and usually from 5 to 20 tumours are thus removed. As many as 36 were removed from one uterus and yet a very respectable organ was left. The appearance of the uterus after such proceedings is often very ragged and unpromising. At first, he says, "I was often worried by the unshapely appearance of the organ I was trying to save, but I soon learned that the uterus after careful trimming of its redundant tissue, and subsequent careful suture, continued to contract and assume a more normal shape up to the very close of the operation; and the examination of such uteri, months and years later, has convinced me that Nature continued to mould these organs into shapes that were not far from normal." Among other conclusions drawn from his cases by Dr. McCosh are, (1) that myomectomy is possible and advisable in the great majority of cases of fibroid tumours in young women; (2) that in performing the operation fear of hæmorrhage should be cast aside, and bold and rapid methods should be adopted; (3) that the operation is attended with the same danger to life as is hysterectomy; and (4) that the ultimate results as regards menstruation, pain, and pregnancy are satisfactory.

[1] Medical News, September 27.

